# Contextualizing Medications for Opioid Use Disorder and Peer Support Service Provision in the Probation System with Implementation Science

**DOI:** 10.21203/rs.3.rs-3607705/v1

**Published:** 2023-11-23

**Authors:** Augustine Kang, Amelia Bailey, Siena Napoleon, Rosemarie Martin

**Affiliations:** Brown University School of Public Health; Brown University School of Public Health; Brown University School of Public Health; Brown University School of Public Health

**Keywords:** Opioid Use Disorders, Peer Support, Treatment Provision, Implementation Science

## Abstract

**Background:**

Medications for opioid use disorder (MOUD) is an evidence-based approach that reduces opioid-related mortality, particularly among criminal legal-involved persons who are at increased risk of adverse outcomes related to OUD. Implementing evidence-based approaches in the context of probation settings requires an in-depth understanding of specific contexts to improve intervention efficacy and effectiveness. Here, we use the Exploration, Preparation, Implementation, and Sustainment (EPIS) framework to understand implementation contexts for MOUD provision to the probation setting.

**Methods:**

In-depth individual interviews were conducted with key programmatic stakeholders (treatment providers and probation staff involved in service provision for people on probation). The study examined stakeholder perspectives regarding MOUD and Peer Support Service (PSS) implementation among people who are involved in community supervision. Deductive and inductive thematic analysis was conducted, and subsequently the codes, subcodes, and themes were mapped onto the EPIS framework to better understand implementation contexts.

**Results:**

We deduced key inner, outer, and bridging contexts that shape treatment service provision for individuals with OUD who are on probation. Inner contexts include a strong organizational climate that supports MOUD implementation and enthusiasm for peer support services. Outer contexts include difficulty navigating insurance among providers, treatment costs, and systemic stigma towards MOUD. Bridging contexts include a lack of collaboration/communication between relevant agencies (e.g., probation and courts).

**Conclusions:**

Findings indicate the implementation is complex and requires a coordinated effort between correctional systems, probation agencies, and community-based treatment providers.

## Background

Individuals with opioid use disorder (OUD) involved with the criminal legal system are at increased risk for opioid-related mortality upon release from incarceration (1). More than half of incarcerated individuals have a substance use disorder and approximately 23% have OUD (2). Medications for opioid use disorder (MOUD) are an evidence-based treatment for OUD that reduces the risk of overdose and adverse health outcomes (3), and while the systematic provision of MOUD in correctional settings is not the norm, prisons and jails have begun implementing OUD treatment to address the overdose crisis. Diverse strategies to improve OUD treatment engagement and retention for those in the criminal legal system are needed, particularly for improving transitions across systems of care (e.g., from controlled correctional settings to community settings).

Recovery support services are a range of non-clinical services and supports that when combined with MOUD help individuals initiate support and maintain recovery from SUD. Peer support services (PSS; where individuals who have experienced similar challenges provide guidance to others) are a form of recovery support services that help to retain vulnerable populations in medical care and treatment for OUD within a variety of settings (4). Peer support for criminal legal involved people can lead to increased retention in treatment programs and reduced rates of recidivism (5, 6). However, evidence examining the utility of PSS among individuals with OUD involved in community corrections has not been thoroughly explored.

The Exploration, Preparation, Implementation, and Sustainment framework of implementation guides the effective implementation of novel practices through the identification of key factors and processes (7, 8). Broadly, the phases within the framework identify/define the problem to be addressed, identify sources/stakeholders, and develop a plan for the implementation. Importantly, EPIS includes consideration of inner, bridging, and outer contexts. Inner contexts are internal factors within the organization that can impact the program, such as organizational culture, available resources, and existing processes. Bridging contexts are external factors that connect the organization to its environment, such as stakeholders, partners, and regulatory environment. Outer contexts are external factors that influence the project, such as technological and societal changes. EPIS has been applied in multiple settings including within correctional institutions offering MOUD (9–11). To better understand how to prepare criminal legal and medical systems for programs to treat OUD in community supervision settings, we examined the contextual factors of MOUD provision in probation settings using the EPIS framework.

## Methods

A purposive sample of probation staff and healthcare providers (n = 10) working in Northeastern United States were recruited in 2021 for the study. Participants have a key organizational role in either (1) the probation system (which includes probation officers and supervisors) or (2) a community treatment program (e.g., medical staff with clinical roles). Semi-structured qualitative interviews were conducted with participants to gather information about their perceptions and experiences related to the provision of MOUD and PSS to individuals on probation. Interviews were conducted on Zoom and lasted 50–80 minutes. The interview guide consisted of open-ended questions that explored participants’ understanding and perspectives of the healthcare needs of individuals on probation and/or community supervision and barriers/facilitators to accessing healthcare services. The interviews were transcribed verbatim and then analyzed using inductive and deductive thematic analysis. Two researchers independently coded the transcripts and met to discuss and reconcile any discrepancies in their coding. The final set of codes and themes were reviewed by a third researcher to ensure the credibility and validity of the analysis. We then categorized responses in accordance with the (1) inner, (2) outer, and (3) bridging contexts of EPIS.

## Results

Table 1 presents a summary of results.

### Inner contexts

Participants shared that organizational change in the probation department was incremental and initiated by top-down leadership decisions. The hierarchal relationship between probation leadership and staff was reinforced by trust, where staff were compliant with organizational changes they felt were “*appropriate for [the] department to succeed and do better” [Participant 1005].* The probation staff felt this trust was mutual and that leadership also listened to officers when they shared their thoughts.

Participants also shared that leadership-led organizational change influenced staff views on OUD treatment over time, particularly in influencing the adoption of a treatment-centered approach for clients with OUD. Participants believed that clients would receive better OUD treatment and retain in care:

“…they’ve taken the stance that they are aware that re-incarcerating somebody for something that may not be the most significant thing is not helpful for them in general, so I have noticed…what they’re doing is re-referring them to treatment.” [Participant 1010]

Peers play an important and unique role due to their ability to connect people with OUD treatment and resources and to share experiences and characteristics with clients (i.e., recovery and criminal legal histories). Participants expressed enthusiasm and a positive outlook on the use of peer support:

“I think there’s a significant value in a peer support or relationship with an offender immediately upon release, because we do have a lot of overdoses immediately upon release. I think that’s really important … they’re really successful and vital and helpful. And they’re the boots on the ground that do a lot of real community work? And hands on hands work with people who need support and help. And it’s the real work.” [Participant 1003]

However, several participants mentioned staff stigma towards people with OUD, and MOUD, and staff resistance to adopt new practices for OUD treatment as barriers. Participants felt staff, especially those who had been with the organization longer, still held the belief that “*methadone is just a poor substitute for the heroin or fentanyl.” [Participant 1006]*

### Outer Contexts

Outer context results centered on respondents’ experiences as community-based MOUD providers and probation officers with a focus on health insurance, costs, and systemic stigma.

Community-based MOUD treatment providers and patients face unique challenges to pay for MOUD services: when incarcerated, the individual’s health insurance benefits are suspended, and health insurance costs are covered by the state. Discharge planning staff assist individuals who are leaving incarceration to enroll in Medicaid (if qualified) to re-initiate insurance. We learned about challenges associated with paying for MOUD while in the community and navigating the costs of insurance coverage:

“For methadone treatment specifically… they’re just kind of in this weird bracket where, they make this a little too much to qualify for Medicaid, but they’re not really making enough to really support a monthly premium and then reaching those deductibles and things like that….” [Participant 1008]

Even with insurance and other forms of payment assistance, patients are sometimes forced to choose between paying for MOUD or other expenses, such as food, housing, or transportation:

And he or she will say, their money is tied up every week on paying a Methadone provider….So all your money has to go to your Methadone provider, right, at the time, so you’re even not able to pay for other things. And there was times that I would feel they’re charging way too much money for this. [Participant 1004]

With the shift towards adopting telehealth treatment provisions, there were expressed concerns about the lack of accessibility to patients:

“Just overall the system’s archaic and that’s a huge barrier to successfully integrating technology into supervision. Phone supervision was okay except you’re not getting as good of a feel for what’s really going on with someone.” [Participant 1007].

Systemic stigma towards OUD and MOUD may present challenges to be considered in MOUD service provision among the population:

“There might be stigmas involved. If maybe the community will block a center that provides methadone, a methadone treatment center. There could be individual stigmas, parents just in denial thinking that if their kid gets treatment and they’re seen in one of the centers, they’re going to be stigmatized. So I could see just the regular community blow back, not in my backyard mentality…” [Participant 1003]

### Bridging Contexts

Bridging contexts focused on the connection between agencies (e.g., between probation and courts) that hinders treatment planning and barriers associated with the use of PSS.

While there was optimism for the use of PSS, some practical problems relating to the logistics of implementation were mentioned by some participants:

..that’s the thing overall with addictions. If somebody’s ready to accept help, it has to be available in a short time frame. If…make them wait weeks, by the time it comes around, “Oh, my problem’s not as bad as I thought”… So, again, that responsivity that people have to be ready and available within a pretty short time frame. [Participant 1005]

And,

“…barriers would just be finding them because I can never find them. Like I said, housing is a huge problem in [state] and there are so many homeless people right now. Like I said, phone numbers change all the time. It’s a little bit crazy” [Participant 1007]

A lack of communication/coordination between government agencies may adversely affect the care provision:

“I think there’s a lot of systematic issues that our department is not necessarily responsible for… the biggest one is the judiciary and the Attorney General’s office. They seem like they’re an entity among themselves and there is just not a lot of collaboration” [Participant 1003].

## Conclusion

Overall, our findings identify inner and outer context factors that may facilitate and impede adoption of MOUD programming and highlight bridging context factors that need to be addressed to improve service delivery.

Results indicate a need for further collaboration between criminal legal agencies (e.g., probation agencies and courts). Such a collaboration is crucial in providing substance use treatment to individuals in community supervision; Probation agencies are responsible for monitoring and enforcing conditions of probation, while courts navigate circumstances surrounding terms of release, violation, and revocation. Together, probation agencies and courts can ensure that individuals on probation have appropriate access to needed substance use treatment, and that treatment is appropriately monitored and supported as a condition of probation. This collaboration may help to reduce recidivism and improve overall public safety.

Our finding of staff stigma toward individuals with OUD and MOUD is consistent with previously published evidence highlighting stigma among healthcare workers in prisons and jails (12). Stigmatizing beliefs among healthcare workers and probation staff can shape individual’s preferences for receiving MOUD treatment, and hence represents an opportunity for intervention to improve MOUD uptake (13, 14). Peer support services can provide a sense of community and belonging for individuals when they transition across systems and work to overcome addiction. Additionally, PSS can provide practical assistance such as help with finding housing and employment, as well as emotional support and encouragement upon return to substance use. Our study shows that while there is support for PSS, there are practical and logistical considerations to keep in mind in ensuring the connectedness between peers and their clients.

Navigating insurance payments for healthcare services provided to individuals who are incarcerated can be challenging for healthcare providers. One major difficulty is that many incarcerated individuals are covered by government-funded insurance programs, such as Medicaid or Medicare, that have reimbursement rates and requirements different from private insurance. Furthermore, many incarcerated individuals have complex health needs and require specialized services, such as mental health or substance abuse treatment, that may not be covered by their insurance or may require pre-authorization. These difficulties can lead to financial and/or administrative burdens for healthcare providers and may also impact the quality and availability of healthcare services for criminal legal involved individuals.

The implementation of evidence-based care for OUD, including the use of MOUD, for individuals involved in the criminal legal system is a crucial to addressing the opioid epidemic and reducing recidivism. However, the implementation itself is complex and requires a coordinated effort between prison systems, probation agencies, and community-based treatment providers. Collaboration between these entities is essential to ensure continuity of care for individuals transitioning across systems of care.

## Figures and Tables

**Table 1:** Illustrative quotes to contextualize the inner, outer, and bridging contexts.

Inner contexts	
Strong hierarchical structure	*“Well, again, it’s all an issue of communication in an organization. Sometimes we don’t feel like they always hear us where things don’t get filtered up as high as they should that we might share information with our supervisor. And the supervisor might share it with a level above, but then you question how far up it goes beyond that.”* [Participant 1005]
	*“It still tends to be a hierarchical situation because corrections does have kind of a semi-military attitude… sometimes the hierarchy can get in the way of communication, especially up.”* [Participant 1005]
Trust in organization leadership	*“I would say i trust our team… They try to think of things [to] help in outreach and stuff like that. I think as a whole, change is just hard in general, more for frontline workers, because either we have people who have been there a really long time, so they’re just so used to doing something in a particular way.”* [Participant 1008]
	*“We hear it with staff meetings all the time from our supervisor that administration wants us to start working with individuals using [referral to MOUD and other services]… They really are pushing us to do it.”* [Participant 1006]
Positivity towards MOUD programs	*“I think…[the organization’s mission regarding MOUD] would be an individualized approach to… connect people to medicated assisted treatment in a way that best serves their needs. So, essentially a patient centered approach to opiate use disorder.”* [Participant 1009]
	*“As far as the inmates inside, they’ve done really, really well with getting them on MAT if they request it and trying to keep them connected when they are released because that’s when they’re the most vulnerable… the inside prison program i think has been pretty successful.”* [Participant 1007]
Resistance to adopt new practice around OUD	*“When people are locked into what they do then they have a hard time seeing themselves doing something different.”* [Participant 1005]
	*“if my superior comes to me and says, i’ve run a courthouse operations here. So if they come and say, well, starting tomorrow, you’re going to do things this way. And it doesn’t make any sense, or i don’t really understand why you’re doing it, i just want to obviously hear the rationale behind the changes that you’re going to be making* [Participant 1004]
Importance of peer support	*“I think that there needs to be… some value placed with the support. And maybe it’s not a counselor, right? So it may be… if the persons on MAT they don’t have an assigned counselor, but at least they have an assigned peer support…”* [Participa nt 1004]
	*“So I’m a heavy believer in if you’re going to get better, who better to show you the way then someone’s been there before?”* [Participant 1003]
**Outer contexts**	
Lack of transportation as a barrier to MOUD	*“I hear a lot from patients that the reason that they fall off treatment is transportation. That’s one of the main ones that I hear often…”* [Participant 1009]
	*“Be accessible. Be where they’re at. Transportation is a problem for a lot of people. So just be visible to them and I think that would be really successful.”* [Participant 1007]
Stigma as a systemic barrier	*“I think that I wish and hope that the community, as things develop and education’s more aware… I’d just like to see the stigma de-stigmatized. it’s heart-wrenching when they come in and they’re either like, ‘I’m not like the rest of your patients,’ or the other day, we had somebody who was like, ‘Well, I don’t really want to come in every day because I might know somebody. ‘”* [Participant 1008]
	*“[Some probation officers] think it’s a crutch. it’s another drug that they’re using and they should just not be on anything.”* [Participant 1006]
Bridge to the community treatment is important	*“We do a lot of medical stuff… We provide a lot of case management services… to help coordinate with outside providers, help set them up with different appointments. We coordinate with legal services and… as much to our ability we’ll help with like housing applications, and just even like job applications, resume building stuff…”* [Participant 1010]
	*“Well, I think it has to be a coordinated effort. So, let’s say you have somebody who’s released from the prison and they’re on methadone or Suboxone. So, if they get out in the community, but there aren’t supports there, let’s say they don’t have housing, they don’t have a job, they don’t have healthy social supports. Eventually, they just might say, ‘Forget about it,’ and relapse.”* [Participant 1005]
**Bridging contexts**	
Positive outlook of peers	*“…I’ve had some people over the years especially when I was in [city] because [local treatment center] drop-in center with the recovery coaches was right there downtown. So, I had more people when I was working out of the [city] office who had recovery coaches and would talk about it. And generally, those that chose to do it had a positive experience.”* [Participant 1001]
	*“…I honestly encourage every client you have to try to get involved in care recovery services in some way or another because I just think it’s really helpful to be able to have that other additional support, somebody that knows like what you’re going through or has gone through something very similar.”* [Participant 1010]
Barriers to implementing peer program	*“With the DOC population, a lot of them don’t… I don’t even have phone numbers for.. I have so many homeless people on this caseload and their phone numbers change all the time. So technology could be a problem for some.?”* [Participant 1007]
	*“A difficulty would be if you didn’t have any funding, right? That would make it more difficult. Just, not having buy in or not having people believe in the success or the positive benefits of your program. Not having the right people supported and being able to get the message out and highlight the benefits of the program versus the negative.”* [Participant 1004]
Poor communication with external government agencies	*“Sometimes I will have a release signed and I will call the counselor and call the counselor and call the counselor and then re-fax the release and finally I might get a fax back… But a lot of counselors, they’re probably leery [of] probation and then they don’t want to talk to us. Especially if someone is using, they don’t want to get that person in trouble… I think breaking down those barriers would be super helpful.”* [Participant 1007]
	*“…it’s all an issue of communication in an organization. Sometimes we don’t feel like they always hear us where things don’t get filtered up as high as they should that we might share information with our supervisor. And the supervisor might share it with a level above, but then you question how far up it goes beyond that.”* [Participant 1005]
Hindrance of work by judicial system	*“…the judicial branch seems to hinder our ability to do our jobs the way it should be done”* [Participant 1003]
	*“There’s disjoint-ment between the enforcement and the judge will demean probation in court. They’ll yell at probation in court. They’ll roll their eyes. There’s no consistency between judge and… sentencing. it’s very disheartening to be marginalized by the judiciary when we’re out there, busting our asses and putting our lives at risk, you know?”* [Participant 1003]

## Data Availability

The datasets used and/or analyzed during the current study are available from the corresponding author on reasonable request.

## Abbreviations

OUD: opioid use disorder
MOUD: medications for opioid use disorder
PSS: peer support services
EPIS: Exploration, Preparation, Implementation, and Sustainment framework
